# Reduced Metabolism in Brain “Control Networks” following Cocaine-Cues Exposure in Female Cocaine Abusers

**DOI:** 10.1371/journal.pone.0016573

**Published:** 2011-02-23

**Authors:** Nora D. Volkow, Dardo Tomasi, Gene-Jack Wang, Joanna S. Fowler, Frank Telang, Rita Z. Goldstein, Nelly Alia-Klein, Christopher Wong

**Affiliations:** 1 National Institute on Drug Abuse, Bethesda, Maryland, United States of America; 2 National Institute on Alcohol Abuse and Alcoholism, Bethesda, Maryland, United States of America; 3 Medical Department, Brookhaven National Laboratory, Upton, New York, United States of America; Chiba University Center for Forensic Mental Health, Japan

## Abstract

**Objective:**

Gender differences in vulnerability for cocaine addiction have been reported. Though the mechanisms are not understood, here we hypothesize that gender differences in reactivity to conditioned-cues, which contributes to relapse, are involved.

**Method:**

To test this we compared brain metabolism (using PET and ^18^FDG) between female (n = 10) and male (n = 16) active cocaine abusers when they watched a neutral video (nature scenes) versus a cocaine-cues video.

**Results:**

Self-reports of craving increased with the cocaine-cue video but responses did not differ between genders. In contrast, changes in whole brain metabolism with cocaine-cues differed by gender (p<0.05); females significantly decreased metabolism (−8.6%±10) whereas males tended to increase it (+5.5%±18). SPM analysis (Cocaine-cues vs Neutral) in females revealed decreases in frontal, cingulate and parietal cortices, thalamus and midbrain (p<0.001) whereas males showed increases in right inferior frontal gyrus (BA 44/45) (only at p<0.005). The gender-cue interaction showed greater decrements with Cocaine-cues in females than males (p<0.001) in frontal (BA 8, 9, 10), anterior cingulate (BA 24, 32), posterior cingulate (BA 23, 31), inferior parietal (BA 40) and thalamus (dorsomedial nucleus).

**Conclusions:**

Females showed greater brain reactivity to cocaine-cues than males but no differences in craving, suggesting that there may be gender differences in response to cues that are not linked with craving but could affect subsequent drug use. Specifically deactivation of brain regions from “control networks” (prefrontal, cingulate, inferior parietal, thalamus) in females could increase their vulnerability to relapse since it would interfere with executive function (cognitive inhibition). This highlights the importance of gender tailored interventions for cocaine addiction.

## Introduction

Gender differences in the vulnerability for substance use disorders including cocaine dependence have been reported [1]–[3]. For example, females are 3–4 times more likely than males to become addicted within 24 months of first cocaine use [4]. Also clinical studies report that women seek treatment for substance use disorders earlier than men [5]–[8] and that they don't recover as quickly from cocaine abstinence as their male counterparts [6]. In turn, others have reported that male cocaine abusers appear to transition from abstinence to relapse and viceversa at a significantly greater rate than females [9].

The mechanisms underlying the reported gender differences in the vulnerability for cocaine use disorders are likely to be multiple including differences in pharmacological sensitivity to cocaine [1] and in reactivity to cocaine-cues and to stress [10]. The differences in reactivity to cocaine-cues are particularly relevant since they drive continued cocaine use [11]. However, few studies have evaluated gender differences in cocaine-cue reactivity and the results are inconclusive. At least two studies have reported greater reactivity in women than in men [12], [13] one showed the opposite [14], one showed no differences [15] and one showed differential sensitivity to different cue-induced measures [16]. The reasons for the discrepancies are likely to be multiple including heterogeneity of cocaine abusing populations, methods used to elicit craving and the period during the menstrual cycle when the measurements were done. Specifically, imaging studies have shown greater reactivity to reward during the follicular than the luteal phase of the menstrual cycle [17]. Also subjective reports may not necessarily reflect brain reactivity in all subjects since deficits in interoception in cocaine abusers may interfere with awareness of craving [18]. Here we test the hypothesis that the brain of female cocaine abusers when tested in the midfollicular phase of the menstrual cycle is more reactive to cocaine-cues than that of male cocaine abusers.

To test this hypothesis we compared the regional brain metabolic changes induced by cocaine-cues between female and male cocaine abusers. We used Positron Emission Tomography (PET) and 2-deoxy-2[^18^F]fluoro-D-glucose (^18^FDG) to measure brain glucose metabolism (marker of brain function) [19] in 16 males and 10 female active cocaine abusers that were part of two studies that assessed the effects of stimulant drugs and cognitive strategies to control craving [20], [21]. Subjects were tested twice; once while watching a neutral video (nature scenes) and once while watching a video of cocaine-cues (repeating scenes of people taking cocaine). The cocaine-cues video used was one we had previously shown induced significant increases in striatal DA, which was an effect associated with increases in drug craving [22]. Cocaine craving was measured using analog self-reports of craving and a brief version of the Cocaine Craving Questionnaire (CCQ) that evaluates current cocaine craving [23].

## Results

### Behavioral Measures

Self-reports of craving were increased by the cocaine-cues video; the repeated factorial ANOVA showed a significant condition effect (F = 5, df 1, 24; p<0.03) but the interaction was not significant (F = 2, p<0.17). Females tended to show larger increases (3.4±2 vs 5.8±3) than males (3.0±3 vs 3.7±3) but the gender difference was not significant. Similarly the cocaine-cues increased the scores on the CCQ both in females (29±9 vs 38±12) and males (30±12 vs 34±15) but the gender difference was not significant.

### Absolute brain metabolism

Whole brain metabolism showed a significant cue by gender interaction effect (df 1, 24 p<0.05). In females whole brain metabolism significantly decreased (−8.6%±10; p<0.03) with the cocaine-cues video exposure (35.6±4 µmol/100 g/min) as compared to neutral (32.3±3 µmol/100 g/min); whereas in males whole brain metabolism tended to increase (+5.5%±18) with cocaine-cues exposure (36.2±5 µmol/100 g/min) as compared to neutral (34.9±5 µmol/100 g/min) but in the males this effect was not significant.

The SPM analysis on the absolute metabolic images in the females showed significant differences for the uncorrected threshold p_u_<0.005 for decreases in right midbrain (MNI coordinates for x (left to right), y (anterior to posterior) and z (top to bottom) of 3, −9, 15), left anterior cingulate (BA 24; MNI coordinates −14, 13, 30) and left lateral orbitofrontal cortex (BA 47; MNI coordinates −51, 24, −5). In males SPM did not detect any significant difference in absolute metabolism even when the threshold was reduced to p_u_<0.05. The cue by gender interaction showed that the differences were significant for p_u_<0.005 for greater cues-induced decreases in the females in anterior cingulate gyrus (BA 24; MNI coordinates −14, 13, 30 and BA 32 −18, 16, 38) and in left inferior frontal gyrus (BA 47, MNI coordinates −45, 39, −11).

### Normalized metabolic measures

The SPM results were very similar when daily cocaine use was entered as a covariate than when it was not. Here we present the results for the analysis after adjusting for the amount of cocaine used (daily cocaine use) since this was higher for the males than for the females.

**Figure 1:**
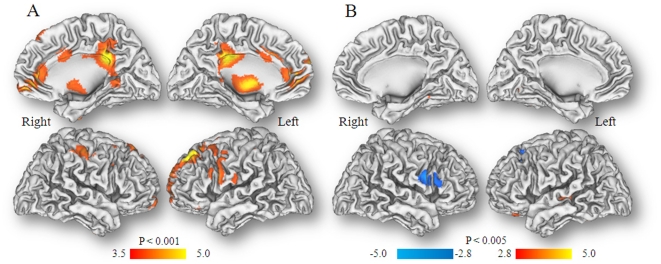
A. SPM results for the “normalized” metabolic images for Neutral vs Cocaine-cues video conditions in the Females. Comparison correspond to Neutral > Cocaine-cues for uncorrected threshold p_u_<0.001 cluster >200 voxels. There were no regions where metabolism was higher during the Cocaine-cues than the Neutral conditions. **B.** SPM results for the “normalized” metabolic images for Neutral vs Cocaine-cues video conditions in the Males. Comparison correspond to Neutral > Cocaine-cues for uncorrected threshold p_u_<0.005 cluster >200 voxels; we used this lower threshold since there were no significant differences for p_u_<0.001. There were no regions where metabolism was higher during the Cocaine-cues than the Neutral conditions.

In the females SPM analysis on the normalized metabolic images (p_u_<0.001) showed decreases in anterior cingulate (BA 24, BA 32), frontal (BA 4, BA 8), posterior cingulate (BA 23,), inferior parietal (BA 40), thalamus (ventro lateral) and midbrain. There were no regions that showed increases in metabolism (Figure 1A, Table 1).

**Table 1:** Clusters where the SPM analysis done on the normalized metabolic images showed significant changes (Neutral > Cocaine-cues) for the female participants (p_c_>0.05, cluster >200 voxels) after covarying for amount of cocaine used (daily use).

Brain Region	BA	x	y	z	T-score	Z-score	Cluster size
Cingulate Gyrus	31	6	−42	30	5.38	4.32	1817
Posterior Cingulate	23	−4	−38	28	5.13	4.17	
Superior Frontal	8	−24	40	48	5.33	4.20	3716
Anterior Cingulate	32	10	50	−4	4.97	4.08	
Anterior Cingulate	24	−10	36	8	4.94	4.06	
Midbrain		2	−8	−14	5.06	4.13	1758
Thalamus	Ventral Post Lateral	−22	−20	4	4.52	3.81	
Inferior Parietal	40	54	−34	56	4,64	3.88	557
Postcentral Gyrus	2	56	−16	52	4.53	3.82	
Precetnral Gyrus	4	64	−12	40	4.36	3.70	

The clusters show the location of anatomical region including Brodmann Area (BA) and the coordinates in the Montreal Neurological Institute coordinates x (left to right), y (anterior to posterior) and z (top to bottom), and the corresponding statistical measures at the voxel level (T and Z scores) along with the cluster size for number of voxels. There where no regions where (Neutral < Cocaine-cues). Note that in the males none of the clusters was significant at p_c_<0.05.

In the males SPM analysis on the normalized metabolic images (p_u_<0.001) showed no significant differences. Reducing the threshold of p_u_<0.005 identified an area that showed increases in the right inferior frontal gyrus (BA 44/45) but this region did not survive the cluster correction for multiple comparison (Figure 1B).

**Figure 2:**
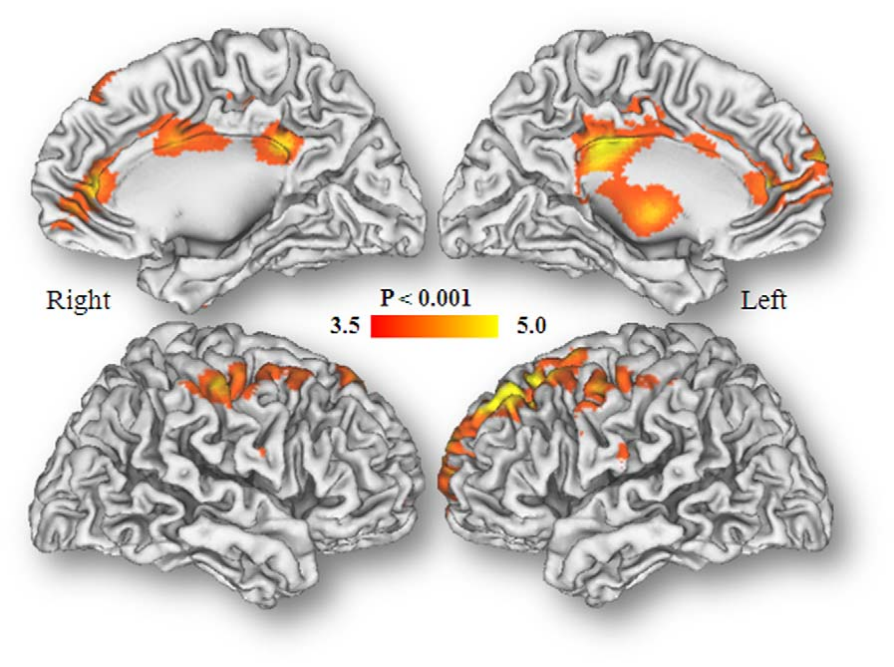
SPM results for the gender by cues (Neutral > Cocaine-cues) interaction on the “normalized” metabolic images. Comparison correspond to Females > Males for uncorrected threshold p_u_<0.001 cluster >200 voxels. There were no regions where males had larger changes than females.

SPM analysis to assess the gender by condition interaction showed that the genders differed significantly (p_u_<0.001). Females had significantly greater decreases than males during the cocaine-cues when compared with neutral in frontal regions (BA 4, BA 6, BA 8, BA 9), in anterior cingulate (BA 24), posterior cingulate (BA 23, 31) and inferior parietal (BA 40) (Figure 2, Table 2).

**Table 2:** Clusters where the SPM analysis showed significant Cues by Gender interaction (Females > Males) for p_c_>0.05, cluster >200 voxels after covarying for gender differences in daily cocaine use.

Brain Region	BA	X	y	z	T-score	Z-score	Cluster size
Middle Frontal Gyrus	6	−28	28	54	5.67	4.47	1185
	8	−22	24	38	5.15	4.18	
	6	−32	14	62	4.97	4.08	
Posterior Cingulate	23	−10	−28	32	5.23	4.23	760
Cingulate Gyrus	31	8	−44	30	4.89	4.04	
Inferior Parietal Lobule	40	58	−30	54	5.19	4.21	625
Middle Frontal Gyrus	6	38	8	62	4.85	4.01	
Precentral Gyrus	4	66	−8	30	4.52	3.81	
Superior Frontal Gyrus	9	−12	62	28	4.92	4.92	823
Anterior Cingulate	24	−10	36	12	4.87	4.02	
Anterior Cingulate	24	10	38	12	4.83	4.00	
Thalamus		−7	−13	5	4.57	3.84	48

The clusters show the location of anatomical region including Brodmann Area (BA) and the coordinates in the Montreal Neurological Institute coordinates x (left to right), y (anterior to posterior) and z (top to bottom), and the corresponding statistical measures at the voxel level (T and Z scores) along with the cluster size for number of voxels. Females showed significantly larger changes in regional metabolism (decreases) than males and there were no brain regions where males had larger changes than females. Note that we report on clusters >45 voxels in order to identify the regions within the large cluster that emerged when we used the preset >200 voxels level.

### Correlations with craving

SPM voxel wise correlation between changes in craving and changes in normalized metabolism were not significant in females or males for p_u_<0.001. Reducing the threshold to p_u_<0.005 showed in males a significant negative correlation in a cluster region (5403 voxels) located in the posterior cerebellum (uvula and culmen); centered at MNI x,y,z coordinates of −36, −65, 24. There were no significant correlations in the females with craving.

## Discussion

The results from this study corroborate our hypothesis of greater brain reactivity to conditioned cocaine-cues in female than in male cocaine abusers even though the self-reported craving responses did not differ between the genders. Females when compared with males showed enhanced brain reactivity (as assessed by changes in brain glucose metabolism) to the cocaine-cues when compared with the neutral condition. The responses were also qualitatively different between genders; whereas in females the cocaine-cues significantly decreased whole brain metabolism in males it was associated with non-significant increases. In addition, the analysis of the normalized metabolic images, which increases the sensitivity to detect regional effects, showed that in the females the cocaine-cues elicited relative decreases in prefrontal cortex, anterior and posterior cingulate gyrus, inferior parietal lobe, thalamus and midbrain whereas in males the only significant difference was an increase in the right inferior frontal cortex (BA 44, 45) that did not survive cluster correction for multiple comparison. The fact that the gender differences in brain reactivity were significant after covarying for the doses of cocaine used indicates that the gender differences were not driven by differences in severity of drug use between the genders.

Traditionally executive control has been ascribed to prefrontal regions, mainly the dorsolateral prefrontal cortex, dorsal anterior cingulate cortex/medial superior frontal cortex and inferior frontal cortex [26]. However studies with functional connectivity have started to identify a more complex set of regions that in addition to prefrontal and cingulate gyrus, include other regions of the frontal cortex, the parietal cortex and the thalamus. Moreover, it has been proposed that these regions are functionally connected into two networks involved with top-down control [27]. Specifically a *fronto-parietal network* comprised of the dorsolateral prefrontal cortex, inferior parietal lobe, dorsal frontal cortex, intraparietal sulcus, precuneus and middle cingulate cortex and a *cingulo-opercular network* comprised of the anterior prefrontal cortex, anterior insula/medial frontal operculum, dorsal anterior cingulate/medial superior frontal cortex and thalamus. It is noteworthy that the gender differences in the brain reactivity to cocaine-cues were all located within one of these two control networks. Thus the deactivation of regions involved with cognitive control with cue-exposure would suggest that top down control may be impaired after exposure to cocaine cues in female cocaine abusers. Indeed, there is evidence of impairment in executive function in cocaine abusers when exposed to conditioned-cues [28]; though to our knowledge no study has as of yet evaluated gender differences in cognitive function following exposure to drug-cues.

In a prior brain imaging study done in healthy controls in whom we exposed participants to food cues and asked them to cognitively inhibit craving we showed that whereas males were able to inhibit limbic brain activation by food-cues, females were unable to do so [29]. If our current findings of metabolic decreases in regions that are part of top-down control networks in female cocaine abusers when exposed to cocaine-cues, generalize to appetitive cues in healthy controls, they could help explain gender differences in the ability to cognitively inhibit limbic activation with exposure to food cues.

In the current study females tended to show greater craving than males when exposed to cocaine-cues but this difference was not significant. Using a similar cocaine-cues video a prior study reported higher levels of craving in females than in male cocaine abusers [12]. Thus in our study we can not rule out the possibility that a larger sample size may have enabled us to document gender differences in craving responses. Regardless, the lack of a correlation between the changes in metabolism induced by the cocaine-cues video and the changes in craving suggest to us that the regional brain responses that we observed with exposure to cues are not the ones underlying the conscious experience of craving but may reflect changes in brain activity that follow the exposure of highly salient stimuli for the cocaine abuser. In this respect it is noteworthy that most research done to understand responses to cues has focused on the experience of craving without recognizing that other processes (mood, executive function) are also likely to be influenced by exposure to conditioned cues.

Our findings differ from prior imaging studies (using fMRI and PET CBF measures) that showed activation of limbic brain regions with exposure to cocaine-cues (scripts constructed to evoke craving or cocaine-cues videos) [30]–[33]. This is very likely to reflect the different temporal sensitivity between the fMRI (measures activity over 5–10 seconds) and PET CBF measures (measures activity over 60 seconds) and that of the PET glucose metabolic measures (measures activity over 30 minutes). Thus the deactivation of regions involved with cognitive control observed with the cocaine-cues video in the females could reflect a long lasting effect that follows the exposure to conditioned-cues in contrast to the fast and short lasting limbic activation from cues exposure.

### Clinical implications

Most clinical studies have focused on the effects of therapeutic interventions to reduce craving [34], [35]. However, therapeutic interventions to weaken the link between craving and drug use have also been shown to be beneficial in cocaine abusers [36]. Indeed, the desire for a drug can be controlled to a greater or lesser extent among drug users and treatment interventions have been shown to decrease cocaine use despite persistent craving [36]. Using imaging we showed in a group of cocaine abusers (predominantly male) that when primed to cognitively inhibit craving in response to the same cocaine-cue video used in this study (but compared to a baseline condition with no stimulation) many of the cocaine abusers were able to decrease subjective experience of craving and to reduce activity in limbic brain regions [21]. Moreover, activity in left inferior frontal cortex (BA 44), which is a brain region implicated in cognitive control [37], predicted the ability to inhibit limbic activity with cue exposures.

In this respect, therapeutic interventions to increase executive function including impulse control may help patients develop coping skills to abstain from using drugs when exposed to cocaine-cues. The findings from this study suggest that these strategies may also benefit by considering gender differences since the mechanism leading to relapse may differ for men and women [9]. Our findings also suggest that there may be gender differences in response to cues that are not necessarily linked with the conscious experience of craving that may nonetheless affect subsequent drug use (i.e., impairing executive function following cues exposures). Indeed a recent study that used real time electronic diary reports showed that in cocaine abusers cue exposures were more frequently associated with drug use than with craving [38].

We recently showed that in cocaine abusers the stimulant medication methylphenidate (MP) interfered with the reduction in metabolism triggered by cocaine cues in cocaine abusers [20] and in a separate study done with fMRI we showed that MP improved executive function and brain activation patterns in cocaine abusers [39]. Also a recent study reported that MP improved stop signal reaction time (SSRT), an index of improved control, in cocaine abusers [40]. Though stimulant medications have not been shown to improve abstinence in cocaine abusers [41] they may be beneficial when coupled with psychotherapeutic interventions that aim to improve executive function and control impulsivity in cocaine abusers.

### Study Limitations

A limitation for this study is the use of conscious awareness of craving as the dependent variable. However, our study does not enable us to assess if genders differ in unconscious responses to conditioned-cues. The studies were done in the midfollicular phase, which is a time at which there may be a greater reactivity to reward and prediction of reward and this reactivity in turn appears to be modulated by estradiol [17]. Indeed, cocaine abusing women when tested in the luteal phase (when sex hormones are higher) have attenuated responses to drug conditioned cues, which were interpreted to suggest that sex hormones have significant effects in regulating brain reactivity to drugs and drug cues [42], [43]. Thus it would have been desirable to quantify the concentration of sex hormones in plasma not only for estrogen and progesterone in the females but also for testosterone in the males. This is relevant since exposure to cocaine could modify the concentration of sex hormones [44], [45]. Moreover, preclinical studies have shown that sex hormones modify the responses to stimulant drugs including cocaine (reviewed [46]) and female cocaine abusers report attenuated subjective responses and less desire to smoke cocaine during the luteal than during the follicular phase of the menstrual cycle [47], [48]. Also the extent to which the greater reactivity to cues extends to other phases of the menstrual cycle and to postmenopausal women requires further evaluation. Moreover though we predict that decreases in metabolic activity in control networks would be associated with impairments in executive functions we did not perform cognitive tests to assess if this was the case (since this was not an a priori hypothesis). Thus studies that evaluate executive function (including impulsivity) after exposure to cocaine cues are necessary to test this. In addition another limitation is the small sample size; yet we were able to detect significant gender differences, which points to the sexual dimorphism in the responses to drug cues in cocaine abusers.

### Summary

This study provides evidence of greater brain reactivity to cocaine cues in female than in male cocaine abusers but no differences in craving responses. Females, but not males showed decreased metabolic activity in brain regions implicated in top-down control network when exposed to cocaine-cues. Further studies to evaluate the cognitive consequences of these responses to cues are necessary to determine if they interfere with inhibitory control and to help guide gender tailored treatment interventions in cocaine use disorders.

## Methods

### Ethics Statement

This study was carried out at Brookhaven National Laboratory and approved by the local Institutional Review Board (IRB of record: Committee on Research Involving Human Subjects (CORIHS); Study #: IRBnet #91581; CORIHS ID #2007-4835; BNL IRB #404) and written informed consent was obtained from all participants.

### Subjects

Twenty six active cocaine abusers (16 M and 10 F) who responded to an advertisement were studied. Subjects fulfilled DSM-IV criteria for cocaine dependence and were active users for at least the prior 6 months (free-base or crack). Exclusion criteria included current or past psychiatric disease other than cocaine or nicotine dependence; past or present history of neurological, cardiovascular or endocrinological disease; history of head trauma with loss of consciousness greater than 30 minutes; and current medical illness. Written informed consent was obtained from all subjects. Table 3 provides demographic and clinical characteristics of participants. Females were studied during the mid follicular phase (7–10 days post last menstrual period).

**Table 3:** Demographic and clinical characteristics of participants.

	MalesN = 16	FemalesN = 10	*P*
Age	43±5	42±8	*NS*
Education	12.7±2	13.0±3	*NS*
Current Smokers	14 of 16	8 of 10	*NS*
Cigarettes per day	12±9	11±6	*NS*
Cocaine Initiation	25±6	22±8	*NS*
Cocaine grams/d	4.0±2	2.4±1	*0.04*
Years abuse	18±7	20±6	*NS*
Age first Rehabilitation	32±5	24±11	*0.05*
CCS	32±13	35±12	*NS*
Body Mass Index (BMI)	26±4	24±4	*NS*

### Behavioral Scales

To assess the subjective experience of craving we used an analog scale (1–10) for self-reports of “cocaine craving” and the brief version of the CCQ [23], which evaluates current cocaine craving on a seven-point visual analog scale. The behavioral measures were obtained prior to (pre) and 30 minutes after (post) initiation of the video. We compared the pre versus the post measures and the corresponding time period for the baseline condition using repeated measures ANOVA.

### Scans

PET scans were conducted with a whole-body, high-resolution positron emission tomograph (Siemens/CTI ECAT HR+, with 4.6×4.6×4.2 mm NEMA (National Electrical Manufacturers Association) using ^18^FDG. Details about the methods for scanning have been published [24]. Briefly, a 20 minutes emission scan was started 35 minutes after injection of 4–6 mCi of ^18^FDG. Arterialized blood sampling was used to measure ^18^FDG in plasma. Subjects were scanned on separate days, once while watching a video of nature scenes (neutral video) and once while watching a video that portrayed subjects smoking cocaine (cocaine-cues video). Videos were started 15 minutes prior to injection of ^18^FDG and continued for 25 minutes after ^18^FDG injection for a total video exposure of 40 minutes. The neutral video featured non-repeating segments of nature stories and the cocaine-cues video featured non-repeating segments portraying scenes that simulated purchase, preparation, and smoking of cocaine [22]. The order of the videos was randomized such that on the first day, half of the subjects were shown the cocaine video whereas the other half were shown the neutral video.

### Image and data Analysis

The data were analyzed using Statistical Parametric Mapping (SPM2) [25]. The SPM analyses were performed both on the absolute metabolic images and on metabolic images that were normalized to whole brain metabolism, which increases the sensitivity to regional effects. The images were then spatially normalized using the PET template provided in SPM and subsequently smoothed with a 16 mm isotropic Gaussian kernel. We used a repeated (neutral vs cocaine-cues) factorial (male vs female) contrast ANOVA analysis for comparison. Since the groups differed in the amount of cocaine used (daily doses of cocaine), which was greater for the males than the females we entered daily cocaine daily use as a covariate in the SPM analyses. Voxel wise correlations were performed to assess the relationship between changes in metabolism and changes in craving separately for the males and females. Significance was set at p<0.001 (uncorrected, >200 voxels) and only regions that survive corrections for multiple comparisons (p_c_<0.05) are reported as significant unless otherwise specified. This was because for the comparisons of the absolute metabolic images there were no significant differences at p<0.001 so we lowered the threshold to p<0.005. Similarly for the comparisons on the normalized metabolic measures in the males there were no significant differences at p<0.001 so we lowered the threshold to p<0.005. Statistical maps were overlaid on an MRI structural image.
